# A Japanese boy with double diagnoses of 2p15p16.1 microdeletion syndrome and *RP2*-associated retinal disorder

**DOI:** 10.1038/s41439-021-00178-2

**Published:** 2021-12-17

**Authors:** Kazuki Yamazawa, Kenji Shimizu, Hirofumi Ohashi, Hidenori Haruna, Satomi Inoue, Haruka Murakami, Tatsuo Matsunaga, Takeshi Iwata, Kazushige Tsunoda, Kaoru Fujinami

**Affiliations:** 1 Medical Genetics Center, National Hospital Organization Tokyo Medical Center, Tokyo, Japan; 2 Department of Pediatrics, National Hospital Organization Tokyo Medical Center, Tokyo, Japan; 3 Division of Medical Genetics, Saitama Children’s Medical Center, Saitama, Japan; 4 Division of Medical Genetics and Cytogenetics, Shizuoka Children’s Hospital, Shizuoka, Japan; 5 Department of Pediatrics and Adolescent Medicine, Juntendo University Faculty of Medicine, Tokyo, Japan; 6 Division of Hearing and Balance Research, National Institute of Sensory Organs, National Hospital Organization Tokyo Medical Center, Tokyo, Japan; 7 Division of Molecular and Cellular Biology, National Institute of Sensory Organs, National Hospital Organization Tokyo Medical Center, Tokyo, Japan; 8 Laboratory of Visual Physiology, Division of Vision Research, National Institute of Sensory Organs, National Hospital Organization Tokyo Medical Center, Tokyo, Japan; 9 UCL Institute of Ophthalmology, University College London, London, United Kingdom

**Keywords:** Medical genetics, Next-generation sequencing

## Abstract

2p15p16.1 microdeletion syndrome is a recently recognized congenital disorder characterized by developmental delay and dysmorphic features. *RP2*-associated retinal disorder (*RP2*-RD) is an X-linked inherited retinal disease with a childhood onset caused by a loss-of-function variant in the *RP2* gene. Here, we describe a 14-year-old boy with double diagnoses of 2p15p16.1 microdeletion syndrome and *RP2*-RD. The recurrence risk of each condition and the indication for potential therapeutic options for *RP2*-RD are discussed.

With the advent of genome-wide technologies such as chromosomal microarray and next-generation sequencing (NGS) analysis, awareness that some patients with genetic disorders can possess another genetic aberration has increased. Obtaining the correct diagnosis in the genetic clinic can provide both patients and their families with crucial information about the natural history and prognosis of the condition, therapeutic strategies, the recurrence risk, and so on. In this context, to correctly diagnose multiple genetic conditions, clinicians should abandon the “single disorder” paradigm and be fully aware of the possibility that some patients may harbor several genetic backgrounds^1^. Herein, we describe a patient with mild intellectual disability (ID) and some dysmorphic features coupled with nonsyndromic retinitis pigmentosa (RP). Initially, we were not completely convinced about the diagnosis of microdeletion syndrome, which was obtained via microarray analysis. Further investigations unveiled the existence of another disease-causing sequence variant accounting for RP.

The propositus was a 14-year-old Japanese boy, a single child of nonconsanguineous parents without a notable medical history. Before birth, intrauterine growth retardation had been observed since the third trimester. In addition, his mother felt that fetal movement had been rather reduced. He was vaginally delivered at term with a birth length of 44.9 cm (−1.98 SD), a weight of 2450 g (−1.55 SD), and a head circumference of 31.5 cm (−1.27 SD). After the neonatal period, he failed to thrive due to progressive feeding difficulties and frequent vomiting, which spontaneously improved as he grew further in life. Growth retardation and global developmental delay (DD) were documented in early childhood (Fig. S1). In addition, he was observed to have borderline ID (an intelligence quotient score of 72) with autism spectrum disorder (ASD). He showed distinctive facial features, including mild telecanthus, downslanting palpebral fissures, ptosis, a wide nasal base, a flat philtrum, thick upper/lower lips, micrognathia, and decreased palmar creases (Fig. 1a−d). Notably, he was followed in an ophthalmology clinic due to photophobia and poor visual acuity from the age of 3 years and night blindness from the age of 9 years; thus, he attended a school for the blind. Fundus examination, fundus autofluorescence imaging, optical coherence tomography, visual field tests, and electrophysiological assessments established the diagnosis of RP (Fig. 2a–g). Of note, brain and orbit MRI scans revealed no abnormal findings. It is also noteworthy that he showed catch-up growth (Fig. S1).

**Fig. 1:**
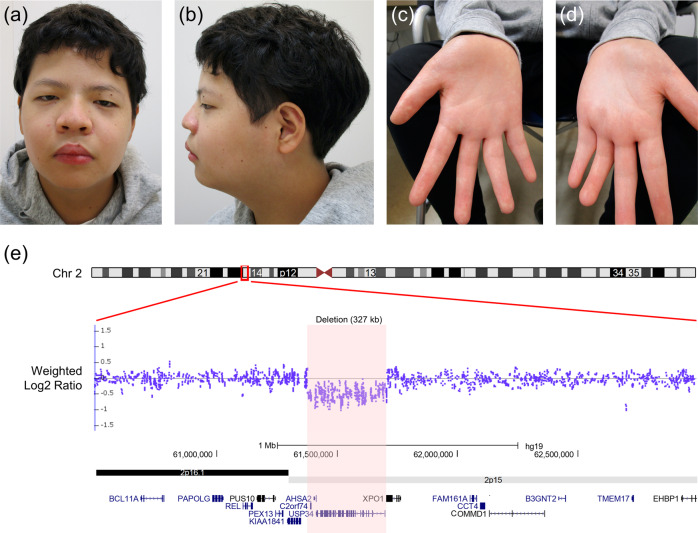
Clinical and molecular findings related to 2p15p16.1 microdeletion syndrome. **a**−**d** The patient at the age of 14 years. Dysmorphic facial features include mild telecanthus, downslanting palpebral fissures, ptosis, a wide nasal base, a flat philtrum, thick upper/lower lips, and micrognathia (**a**, **b**). Decreased palmar creases are also noted (**c**, **d**). **e** Depiction of the deletion in the proband. SNP array analysis showed a 327-kb single-copy loss of 2p15, as highlighted by a light pink rectangle (61,379,351–61,705,869 [GRCh37/hg19]); probes included in the array are represented by blue dots; genes mapped in the region are also denoted. The patient’s parents provided written consent for the publication of the photographs.

**Fig. 2:**
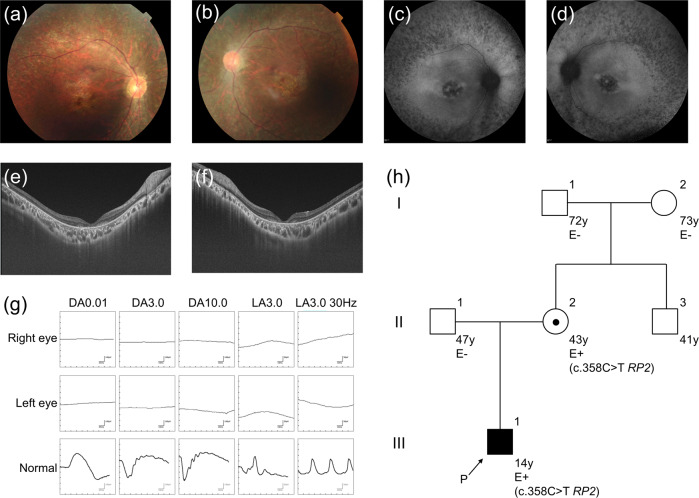
Clinical and molecular findings related to RP2-associated retinal disorder. **a**–**g** Retinal findings of the patient at the age of 14 years. Fundus photography and fundus autofluorescence imaging showed retinal atrophic changes in the entire retina (**a**–**d**). Optical coherence tomography identified a loss of photoreceptor layers in the entire retina (**e**, **f**). Full-field electroretinograms demonstrated undetectable responses under both dark-adapted (DA) and light-adapted (LA) conditions (**g**). **h** Three-generation pedigree. The pathogenic *RP2* variant c.358C > T (p.Arg120Ter) was detected in the proband (III-1) and in the asymptomatic mother with subtle retinal abnormalities (II-2). Females are represented by circles and males by squares; an affected patient with retinitis pigmentosa is shown with a filled symbol, and unaffected individuals are shown with open symbols; a central dot indicates an asymptomatic carrier. An arrow indicates the proband. E+ denotes the above variant carrier in a hemizygous or heterozygous state; E− denotes wild-type (the variant was not detected).

Given the constellation of his multiple anomalies with ID, we proceeded to conduct genetic investigations. After obtaining written informed consent from the patient and parents as well as approval from the local institutional review board, genomic DNA was extracted from peripheral leukocytes. Single nucleotide polymorphism (SNP)-based microarray analysis using a CytoScan HD array (Affymetrix) demonstrated a 327-kb single-copy loss in 2p15 (chr2: 61,379,351–61,705,869, GRCh37/hg19 coordinates) (Fig. 1e). FISH analyses with the BAC probe RP11-17D23 covering the 2p15 region showed heterozygous deletion in the patient but no deletion in the parents (data not shown), indicating that de novo deletion occurred in the patient. Thus, we first suspected a diagnosis of 2p15p16.1 microdeletion syndrome (OMIM #612513), which is a recently recognized congenital disorder characterized by ID, ASD, microcephaly, short stature, distinctive facial features, and structural brain anomalies^2–5^. To date, 40 cases of 2p15p16.1 microdeletion syndrome have been reported in the literature^6,7^. The manifestations of the patient are summarized in Table 1 in comparison with those of previously described individuals with 2p15p16.1 microdeletion syndrome. His manifestations met most of the hallmarks of 2p15p16.1 microdeletion syndrome; however, his retinal findings were not fully explained, although some patients with 2p15p16.1 microdeletion syndrome have been reported to present with optic nerve atrophy^2,4,8^. To determine whether he had another genetic background that resulted in the development of RP, trio multigene panel sequencing analysis targeting 301 retinal disease-associated genes was performed as has been previously reported^9^, and a recurrent hemizygous pathogenic variant NM_006915.3:c.358C > T (p.Arg120Ter) in the *RP2* gene was identified. This variant was also detected in the mother who had subtle retinal abnormalities but was not detected in the maternal grandparents (Fig. 2h), which was confirmed by ultradeep NGS assay (Fig. S2). Thus, double genetic diagnoses of 2p15p16.1 microdeletion syndrome and *RP2*-associated retinal disorder (*RP2*-RD; OMIM #312600) were determined. *RP2*-RD is a rare and severe form of RP with early involvement of the macula, accounting for 1.4% of Japanese patients with inherited retinal disease^9^. The *RP2* variant p.Arg120Ter, which presumably emerged *de novo* in the mother and was passed on to the patient, has been reported to be the most common pathogenic variant causative for *RP2*-RD, potentially as a mutation hotspot^10–12^.

**Table 1:** Summary of the clinical features of the present patient in comparison with previously reported 40 individuals with 2p15p16.1 microdeletion syndrome.

	Present patient	Previously reported individuals
Gender	M	24 M:15 F
IUGR	+	43% (12/28)
Microcephaly	+	65% (26/40)
Short stature	+	43% (12/28)
Intellectual disability	+	100% (40/40)
Language skills delay	+	97% (30/31)
Feeding problems	+	77% (24/31)
Neurodevelopmental delay	+	100% (40/40)
Autistic behavior	+	50% (10/20)
Attention defect	−	54% (13/24)
Hearing loss	−	27% (8/30)
Brain malformation	−	63% (17/27)
Optic nerve hypoplasia	−	17% (4/23)
Retinitis pigmentosa	+	Not yet reported

*M* male, *F* female, *IUGR* intrauterine growth retardation.

Although an increasing number of patients with more than one genetic disorder have been described^1^, reaching a correct diagnosis of multiple underlying conditions remains challenging, particularly when multiple organs are affected. In this case, our team, including pediatricians, dysmorphologists, ophthalmologists, molecular biologists, and genetic counselors with considerable expertise in medical genetics, shared the clinical and genetic characteristics of the patient, thus enabling the determination of the multigenetic mechanism of his manifestations and the establishment of double diagnoses. After chromosomal microarray analysis was adopted as a first-tier test for the ID/DD patient, we added multigene panel sequencing analysis because of a strong suspicion of a second underlying condition for the retinal abnormalities. These accurate diagnoses also allowed us to estimate the genetic risk of each condition; specifically, in a subsequent pregnancy, the recurrence risk of 2p15p16.1 microdeletion syndrome is very low, whereas that of *RP2*-RD is 50% in male offspring. In addition, therapeutic approaches have been developed, and adeno-associated virus-mediated gene augmentation rescued the degeneration of *RP2*-knockout organoids by facilitating morphological preservation and improved rhodopsin expression^13^. Thus, a clear distinction between the origins of ocular and systemic manifestations was crucial to assess the indication for such novel therapies. In terms of therapeutic strategies for *RP2*-RD, notably, the visual prognosis of the mother, a heterozygous pathogenic variant carrier of *RP2*, remains uncertain due to the limited published data.

To conclude, we present a boy with double diagnoses of 2p15p16.1 microdeletion syndrome and *RP2*-RD. When examining a patient with atypical features that do not fit a single diagnosis in the genetic clinic, considering the possibility of a second diagnosis and conducting a further investigation accordingly are of great importance.

## HGV Database

The relevant data from this Data Report are hosted at the Human Genome Variation Database at 10.6084/m9.figshare.hgv.3113

## Supplementary information

Supplementary figures
