# Supplementary material for: A Japanese boy with double diagnoses of 2p15p16.1 microdeletion syndrome and RP2-associated retinal disorder
Source: Hum Genome Var. 2021 Dec 17;8:46. doi: 10.1038/s41439-021-00178-2 (PMC8683409; doi:10.1038/s41439-021-00178-2)
Supplement: Supplementary file 1 — Supplementary figures [file 41439_2021_178_MOESM1_ESM.pptx]

## Slide 1
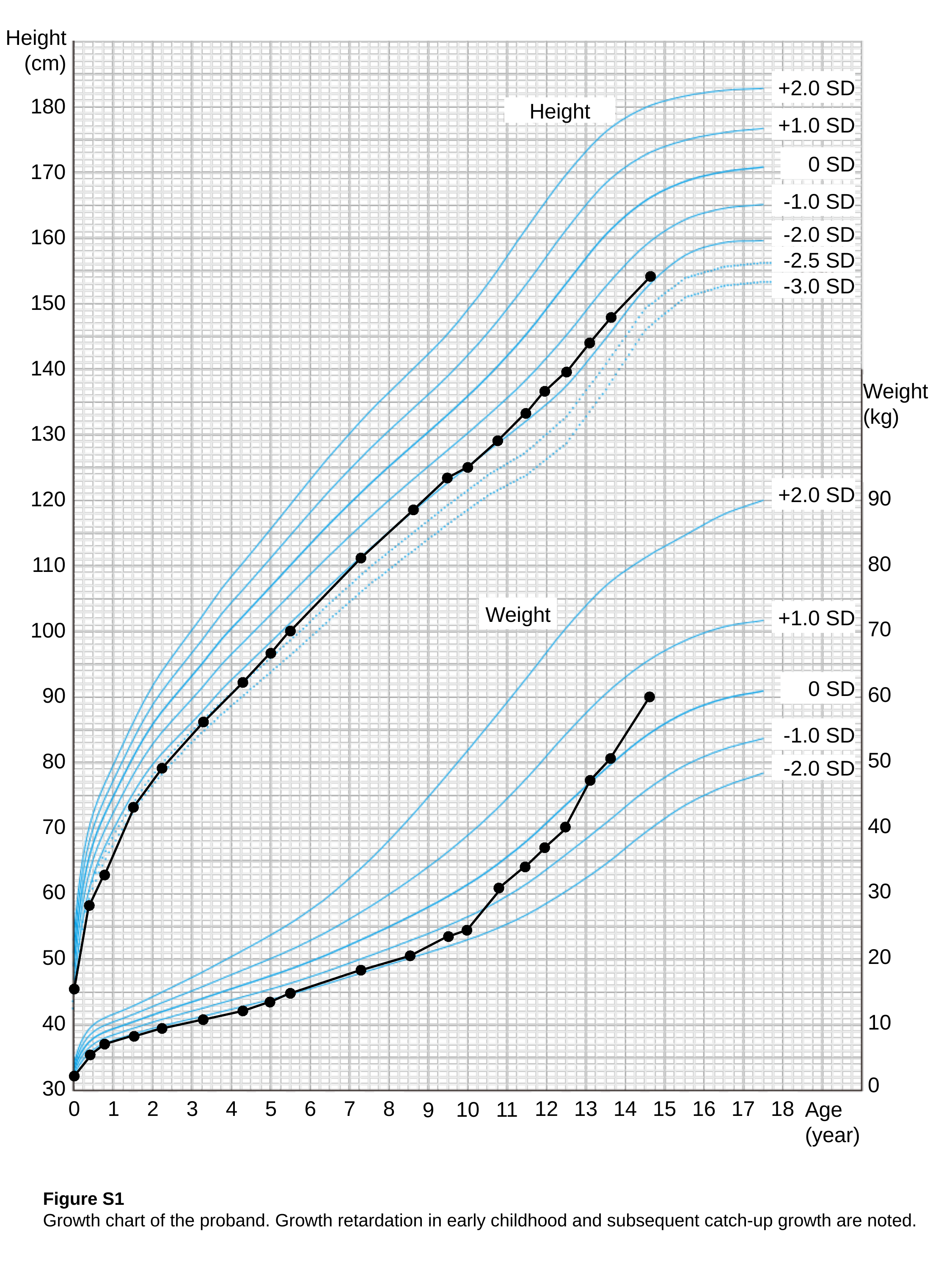

Height
(cm)
+2.0 SD
180
Height
+1.0 SD
0 SD
170
-1.0 SD
160
-2.0 SD
-2.5 SD
-3.0 SD
150
140
Weight
(kg)
130
+2.0 SD
90
120
80
110
Weight
+1.0 SD
70
100
0 SD
60
90
-1.0 SD
50
80
-2.0 SD
40
70
30
60
20
50
10
40
0
30
0
1
2
3
4
5
6
7
8
12
13
14
15
16
17
18
10
11
9
Age
(year)
Figure S1
Growth chart of the proband. Growth retardation in early childhood and subsequent catch-up growth are noted.

## Slide 2
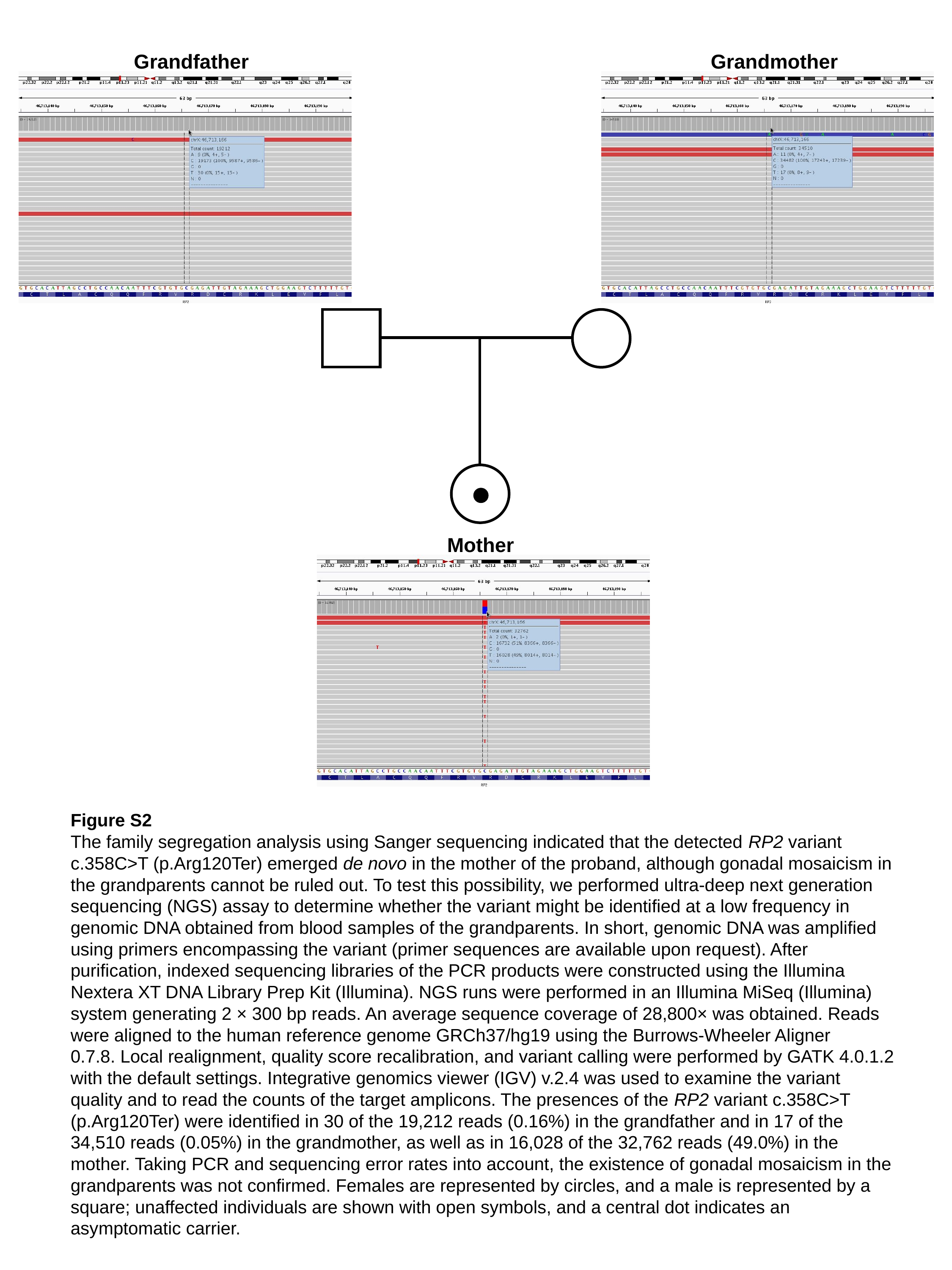

Grandfather
Grandmother
1
1
Mother
Figure S2
The family segregation analysis using Sanger sequencing indicated that the detected RP2 variant c.358C>T (p.Arg120Ter) emerged de novo in the mother of the proband, although gonadal mosaicism in the grandparents cannot be ruled out. To test this possibility, we performed ultra-deep next generation sequencing (NGS) assay to determine whether the variant might be identified at a low frequency in genomic DNA obtained from blood samples of the grandparents. In short, genomic DNA was amplified using primers encompassing the variant (primer sequences are available upon request). After purification, indexed sequencing libraries of the PCR products were constructed using the Illumina Nextera XT DNA Library Prep Kit (Illumina). NGS runs were performed in an Illumina MiSeq (Illumina) system generating 2 × 300 bp reads. An average sequence coverage of 28,800× was obtained. Reads were aligned to the human reference genome GRCh37/hg19 using the Burrows-Wheeler Aligner 0.7.8. Local realignment, quality score recalibration, and variant calling were performed by GATK 4.0.1.2 with the default settings. Integrative genomics viewer (IGV) v.2.4 was used to examine the variant quality and to read the counts of the target amplicons. The presences of the RP2 variant c.358C>T (p.Arg120Ter) were identified in 30 of the 19,212 reads (0.16%) in the grandfather and in 17 of the 34,510 reads (0.05%) in the grandmother, as well as in 16,028 of the 32,762 reads (49.0%) in the mother. Taking PCR and sequencing error rates into account, the existence of gonadal mosaicism in the grandparents was not confirmed. Females are represented by circles, and a male is represented by a square; unaffected individuals are shown with open symbols, and a central dot indicates an asymptomatic carrier.
